# Early stress exposure: Concepts, findings, and implications, with particular emphasis on attachment disturbances

**DOI:** 10.1186/1753-2000-3-24

**Published:** 2009-09-04

**Authors:** Thomas G O'Connor, Mary E Spagnola

**Affiliations:** 1 Department of Psychiatry, Wynne Center for Family Research, University of Rochester Medical Center, 300 Crittenden Blvd, Rochester, NY, 14642, USA

## Abstract

**Background:**

Early intervention and preventive interventions are attracting increasing attention in the child and adolescent mental health field because recent research findings offer new insights into risk mechanisms and because of the growing expectation that they may hold clues to reducing suffering and health cost burdens for society and the individual.

**Methods:**

A selective review of the literature is provided to examine alternative models for understanding the impact of early risk exposure and how these findings may be translated to intervention and prevention; we pay particular attention to the role of child-parent attachment relationship quality as a major potential source of risk or protection.

**Results:**

In this qualitative review, we conclude that sharply contrasting models for understanding early exposure to risk have not been adequately empirically examined in human work. In the case of attachment disturbances, one good context for studying early risk and intervention, sizable questions remain about conceptual models and assessment practices.

**Conclusion:**

Implications of these findings, and limits of the findings, for existing conceptual models of child and adolescent psychopathology and clinical practice are highlighted.

## Review

A fundamental hypothesis in the child and adolescent mental health field is that early interventions and preventive interventions can reduce the likelihood of mental disorder or diminish the impact of impairment on the individual and his or her family [1,2]. To be sure, preventive intervention, that is, intervention with individuals who do not yet exhibit frank disturbance, has for many years been seen as a major clinical research focus and has led to the development of evidence-based programs for children and their families [3]. What is noteworthy about more recent work in this area is that it has been more explicitly linked with experimental animal studies and putative biological mechanisms that may underlie the effects of both the early risk exposure and the clinical benefits of intervention. That provides exciting new opportunities for "basic" and "applied" scientists, clinicians, and policy-makers to form joint collaboratives to improve the health of children and their families. However, uncertainties remain about how well the neuroscientific bases of early interventions derived from animal studies translates to humans. Our selective review considers some of the more striking findings from research on early exposures and bio-behavioral development, and attachment disturbances in particular, and considers what implications they may have for conceptual models, clinical treatment, and what is meant by "evidence-based" practice. We focus particularly on child-parent attachment (and relationship quality more broadly) because it has attracted the most attention and debate.

### Early psychosocial and caregiving risk may have persisting effects on children's health

A range of conceptual models have been proposed for conceptualizing if, and how, early stress exposures or early protective factors may have lasting effect on the individual [4]. In this brief treatment of the topic, we consider two: the cumulative effects or life-course model and the developmental programming model.

A dominant model in developmental and clinical research, which we refer to as the cumulative risk model, proposes that it is the accumulation of risk exposures across setting and time that is most likely to lead to disturbance. Within this approach, no particular impact is attributed to early risk exposure *per se*. Rather, early exposure is thought to have lasting impact on the individual insofar as those early risks are reinforced or maintained by subsequent risk exposure; stated differently, risk exposure that is confined early in development would be expected to have limited long-term impact. An application, particularly for treatment, is that the impact of early stress exposure may be offset by subsequent protective experiences and reduction of risk exposure. There is considerable empirical support for this model, which has also been referred to as a trajectory model (see, [5]). For instance, children's concurrent adjustment may be better predicted from current child-parent attachment than attachment quality assessed earlier in development [6], and maternal depression may not be associated with children's later cognitive development if it is limited to the postnatal period [7]. These kinds of findings underscore the need for careful and ongoing assessment of children's adjustment and risk exposure, and imply that no period in development has disproportionate impact on health and development.

Set against the cumulative risk or life-course hypotheses are alternative models that place particular emphasis on how early risk exposures may confer long-term implications for health and development. One such model is the developmental programming model, which proposes that the organism adapts to environmental input at a particular period in ontogeny, and that this "set point" carries long-term implications for health and disease susceptibility. That is, there is an adaptive quality to the organism's early response or adaptation insofar as it is preparing for its current (and subsequent) environmental demands [8]. This model has emerged from experimental animal work and human research into the developmental origins of health and disease.

A central theme in this article is that we do not yet have adequate data to distinguish clearly between these very different alternatives for understanding the impact of early risk exposure on mental health-related outcomes in children and adults.

Developmental research on psychosocial influences on human development proceeded largely independently from experimental animal studies that had, for many years, demonstrated that *early *exposure to risk (e.g., separation from caregiver) had distinguishable long-term effects on biological and behavioral development. Experimental leverage that was possible in animal studies allowed those investigators to show that there were effects *particular *to early stress exposure, prenatally or postnatally [9,10]. What is not yet clear is how well those findings translated to human development and clinical processes. That is because human studies, with notably few exceptions (see below), do not have leverage for testing the hypothesis that there is something particular to early stress exposure that has a specific impact. The reason for this is that most of the key psychosocial risks for psychopathology in young people - such as poverty, maltreatment, violence exposure - are stable and so exposures are not precisely timed in development. As a result, it is not possible to say that, for example, early maltreatment has an effect on child health and development that is distinguishable from the accumulated exposure to poor care over many years (statistical approaches to resolve this problem are not sufficient).

Reference to animal work on the effects of early exposure to risk is pervasive in writings on human development. That reflects, and has fostered, what may be an over-eager acceptance of the animal data as a model for human research; translation of the animal findings as regards developmental influence of the timing of exposures, for example, is far short of adequate. Fortunately, there are also good reasons for thinking that we are better positioned to evaluate the degree to which the animal data provide a template for, and may be translated to, human development. That may be attributed to several factors. One is the new cohort of studies of ex-institutionalized children that emerged in the human literature about a decade ago. These showed that children whose exposure to deprivation was limited to the early months or years of life nevertheless showed persisting deficits in social and cognitive development even after they had been living in low/normal risk homes for many years [11]; we return to these studies below. A second major influence has been the rise of the developmental origins of health and disease literature [12]. Findings in this area provide compelling evidence that fetal programming may underlie adult diseases such as diabetes and cardiovascular disease [8]. A third stimulus for researching early risk exposure in humans is a variety of improved, accessible, and not cost-prohibitive techniques for assessing biological processes, such as the ready availability of salivary assays and brain imaging techniques to assess structure and function and connectivity in the brain. Although these capabilities provide no particular experimental leverage for testing early exposure effects hypotheses, they do provide a currency for linking biological findings in the animal and human work. The net effect of these trends has been to rejuvenate interest in the hypothesis that early risk exposure may have a particularly strong impact on human bio-behavioral development. To the extent that that is so, there are substantial implications for when (preventive) interventions need to be delivered. The point about the timing of interventions has not yet garnered systematic interest although, as shown in the contrast between the cumulative risk and adaptive programming models above, there are sizable differences in how the timing of interventions for improving (child) mental health might be conceptualized. The point here is not that we do not have substantial evidence for interventions or even early interventions - we do, as shown in the case of the parenting interventions to reduce child disruptive behavior [13] or model early intervention studies [14]. Rather, the point we wish to emphasize is that we do not yet have an evidence base to judge the significance of the *timing *of the intervention; moreover, as noted, existing models for understanding the impact of early risk exposure on behavioral and emotional outcomes lead to dramatically different conclusions about how important early interventions are. It would be impractical to tackle this broad issue in general terms, and so we address this debate in light of recent findings and newly emerging hypotheses concerning the impact of early attachments and disturbances in children.

### Early attachments and attachment disturbances

If we were to evaluate the potential long-term persisting effects of early stress exposure, one place to look for a source of early stress is in the child-parent relationship. Infants are extraordinarily dependent on their caregivers to provide for their physical and psychological needs. It is natural to wonder about whether variation in care quality - from individual differences within the normal range to more extreme forms of abuse and neglect - has temporary and/or lasting impact on the health and development of the child. Experimental animal studies have, for decades, shown that variation in early care has lasting and perhaps permanent influence on the biology and behavior of the offspring [15-18]. Whether or not these findings apply to human development continues to be a matter of uncertainty, both in terms of the effects and the mechanisms involved. There are, for instance, notable biological reasons for questioning generalizations from the animal (especially rodent) to human work [19], and there are some discrepancies even within the animal work [20]. There is intense interest in this issue but, as noted, human studies typically are unable to conclude anything particular about early care from the cumulative impact of poor care because they are confounded in most circumstances.

Probably the strongest analogue in the human or clinical literature are findings on social, cognitive, and behavioral disturbances in children who experienced early institutional deprivation but were later adopted into normal/low risk family environments. The half-dozen or so sets of studies of children adopted into normal/low risk families following institutional rearing are especially powerful because they capitalize on a "natural experiment" in which caregiving adversity was limited to the early months or years of life. We next consider one specific finding from these studies that may have broad implications for theory and treatment, attachment disturbances following early severe deprivation.

A long history of research and clinical reports of young children who experienced institutional rearing [21-23] provided the basis for what eventually became known as reactive attachment disorder (RAD) in the DSM and ICD. The DSM-IV and ICD-10 agree on several features of the disorder, such as it being linked with severe early caregiving disturbances and the existence of two forms of the disorder: the disinhibited ("indiscriminately friendly") and inhibited (withdrawn, hypervigilant) forms. It is not clear how well the minor differences that exist are based in clinical research evidence or would alter assessment or treatment in clinical practice.

Research findings from studies published in the past decade or so substantiate the clinical reports from many decades ago. First, several studies strongly suggest that it is the absence of a consistent caregiver, or the lack of opportunity to form a selective or discriminating attachment relationship, that may have a central causal role in the development of disinhibited attachment disturbance [24-27]. What was not anticipated from the early clinical reports, but is strongly and reliably shown in recent studies, is the huge degree of individual differences. So, for example, approximately 30% of children who experienced 24-42 months of institutional care exhibited a severe disinhibited disturbance in the English and Romanian Adoptee study [28]. That is broadly supported by other studies, which similarly show that severe attachment disturbance is evident in only a minority of children who experienced prolonged institutional care. Tizard and colleagues [29] were able to demonstrate that the attachment disturbance is found in children who experienced institutional care even if there was adequate nutrition and cognitive stimulation, implying that severe attachment disturbance is not an "institutional syndrome" but more likely a disorder associated with caregiver deprivation in particular. That is also the strong conclusion from the important intervention study that showed that improving the child:caregiver ratio and opportunities for regular care was associated with a decrease in disinhibited disturbances [26].

Second, human studies are now showing that the disturbance is persistent. Data from the ERA study, which assessed children at 4, 6, and 11 years of age [28,30] indicated considerable stability in individual differences and a persistence of disinhibited behavior to age 11 years - where it was evident at the earlier assessments. Clearly, the phenotype now used in DSM and ICD need to be re-evaluated in light of the persistence of the disorder and the pre-school-age-oriented nature of the current symptom profile. Progress in understanding the biological bases are attachment disorder behaviors is still quite rudimentary; the strong biological findings reported in the animal literature [31-34] have not been adequately tested. Studies using brain imaging [35] and indicators of HPA axis activity [36] may yield some reliable findings with clinical import. Research into the neuropeptides of social and attachment behavior in humans is in an early stage, and there are lingering questions about how data of this kind are best collected, specifically from where (CNS, saliva, urine) and under what conditions. It is also possible that genetic factors may be found to moderate the effects of early deprivation - that would be the strong prediction given the wide individual differences in outcomes so far reported.

There is well-placed concern in how well the findings from studies of ex-institutionalized children will generalize to other populations of children who experienced severe early caregiving disturbances. Accordingly, it is important that studies of children in the foster care system are scientifically addressing the concept of attachment disorder and key conceptual and clinical debates [37]. Studies of children in foster care reliably show that severe attachment problems - including attachment disorder - are comparatively common and may account for some the quite disproportionate level of care and cost in this population [38].

#### Attachment disorder and attachment theory

It is far from certain that attachment disorder is or should be linked with attachment theory that was developed by Bowlby, Ainsworth [39] and others. Neither DSM nor ICD conceptualizes attachment disorder in terms of attachment theory, and there are some obvious qualitative differences (see [40]). On one hand, attachment disorder is thought to derive from an absence of opportunities for normal attachments to development. Although that is something that figured prominently in the writings of the attachment theorists, and Bowlby's in particular, it has not attracted much attention for some time. Rather, much of the theory concerns the nature and origins of individual differences in qualities of attachment, along a dimension of Security and Insecurity. Whether or not a child developed a Secure or Insecure attachment presupposed that there were adequate opportunities to form a selective or discriminating attachment and, indeed, all of the attachment measures (e.g., the Strange Situation) are conducted on the assumption that there is an attachment relationship between the child and his/her caregiver. In the case of attachment disorder, there is presumption that the child did not have opportunities to form a selective attachment and some doubt about the having have a selective attachment with his/her current caregiver. The implications for what this means for using more conventional attachment assessments is not yet fully understood.

Research that has directly examined the link between attachment disorder symptoms and attachment Security/Insecurity [24,37,41] has consistently found that a sizable percentage of children with disinhibited attachment disorder behavior nevertheless show apparently normal (Secure or Insecure) attachments with the caregiver in standard separation-reunion paradigms or narrative assessments. That initially surprising finding may give further weight to the notion that attachment disorder symptoms, and perhaps especially disinhibited symptoms, reflect a broader disturbance in social approach, avoidance, and fear that are not necessarily detected in conventional attachment assessments. These findings also underscore the important observation from empirical studies that disturbances of the attachment disorder variety may be far more readily observed with a stranger than with the current caregiver.

### Implications for the early experience findings on attachment for intervention and training

The evidence base concerning how and if early adverse rearing has persisting impact on the behavior and biology of the child is not yet adequate; a corollary of that is that we are some way from making strong recommendation about assessment or treatment. In particular, no treatment has been shown to be effective according to the "gold standard" method of evaluation, the Randomized Control Trial (RCT). Neither is there an assessment protocol that has yet been shown to provide optimal data, although several are now under development. That is a disappointing state of affairs for a clinical disturbance that has been recognized in the psychiatric nomenclature for about thirty years. Given these limitations, we offer a brief overview of some of the more promising lines of research that may have clinical import.

#### Assessment

Several sets of assessment strategies have been reported (see [25]). What is common is that they emphasize detailed clinical interview [26,28,37], which is important to circumvent the possibly misunderstood nature of the disturbance that has been promulgated by various sources on the internet and elsewhere. There is also weight placed on clinical data gathering from multiple sources, a standard strategy for any good clinical evaluation, but perhaps especially important in this under-developed area of clinical investigation. Furthermore, a careful and standard assessment of co-morbid conditions is needed given that attachment disorders are often co-morbid with other behavioral and emotional problems and that there has been a tendency to expand the boundaries of attachment disorder to include all manner of "institutionalized" behaviors (e.g., hoarding food). Many clinical investigators also highlight the need for gathering observational data from the child with caregivers and strangers. Although that may be difficult to obtain in many clinical settings, it would offer the kind of scrutiny that has not always attended the clinical diagnosis. There is not yet an evidence-based assessment protocol, but several are in the making, and observational studies may be of some guidance for more immediate use.

One further issue to resolve concerns symptom definition and terminology. The most important of these are the terms "indiscriminately friendly" or "indiscriminate sociability," which are used to describe the disinhibited pattern. Clinical observations suggest that the behavior is not actually "indiscriminate" or "friendly." Specifically, the notion that the behavior is sociable or friendly is incompatible with the experience of the individual to whom the behavior is directed, who is more likely to experience the child's approach as intrusive. Thus, there continue to appear fairly major ambiguities about how the behavioral symptoms are described, and that will only sustain the clinical confusion that now accompanies the diagnosis. The implication is that fairly basic descriptive research is still needed in this area.

#### Treatment

The first point to be made about treatment is that none has been shown to be effective, according to a conventional threshold of what would constitute meaningful clinical evidence. Nonetheless, claims about treatment effectiveness have been made. The trouble with these claims is that they are based on poor and inadequate evidence. It is no longer the case that anecdotal evidence or an individual clinician's report is considered adequate for judging a treatment's success. To be sure, there are limits of RCTs, which are expensive and labor-intensive, and proceed at a slow pace. That means that what might be genuinely good practice may not yet have earned an "approved" rating. In the area of attachment disorder, however, there seems a far greater risk of endorsing treatments that are ineffective or dangerous, and many have been proposed and carried out that are one or both (see [25]. For example, holding therapy and a range of coercive therapies that have been promoted have not been shown to work when subjected to the rigor of an adequate clinical trial, and studies that suggest these programs work are based on inadequately designed clinical evaluations. It is no coincidence that many professional organizations explicitly warn against the use of holding and other coercive therapies.

The lack of an evidence base for treating attachment disorder should not be confused with the sizable evidence base for treating infant-caregiver relationships that are judged to be Insecure. As noted, these are children who had have opportunities to form selective attachment and who show a selective (albeit Insecure) attachment relationship with their current caregiver. Meta-analyses show that the evidence for genuinely attachment theory and research-based treatments to be substantial [42]. These interventions, which focus on improving parental sensitivity to the child's cues and, to a lesser degree, on the factors that inhibit a sensitive caregiver response, have evolved over many years of rigorous clinical evaluation. The demonstration from these projects that improving parental sensitivity yields improvements in the relationship and child functioning may not, however, extend to the child with an attachment disorder and her/his caregiver(s). That is because it is not clear that caregiver sensitivity is substantially impaired. Indeed, there are many clinical examples in which apparently sensitive adoptive parents (as judged, for example, by positive relationships with their own biological children) seem to have little impact on the adoptive (or foster) child's problems in attachment and social relationships. It remains to be seen if parental sensitivity to children with attachment disorder behavior needs to be different or of a higher degree of magnitude, but it seems as likely, given the available evidence, that improvements in parental sensitivity may not yield major improvements in the child-caregiver relationship (although that, too, awaits formal testing).

In the absence of any evidence-based "best practice", we suggest the following general guidelines. Perhaps the most easily recommended treatment strategy is to provide psycho-educational supportive interventions for parents; that is most naturally seen in the case of adoptive and foster parents. There is high likelihood that foster or adoptive parents will have unfounded suspicions about the disorder and their role in it, and these need to be evaluated in light of the available evidence, such as it is. In any event, parents face considerable stresses when raising and a child with attachment disorder behavior. And it is critical to acknowledge, especially for adoptive parents, a sense of disappointment in the relationship not fulfilling what was almost certainly hoped for. That may well fuel feelings in the parent of inadequacy and a position of being de-skilled. The parental sense of not having a relationship with the child that feels or is perceived to be "special" in some palpable way is rightly a focus for clinical intervention.

Part of the psycho-educational intervention may well include strategies to assist the parent in understanding, in a more concrete manner, the nature of the child's difficulties in social relationships, and perhaps especially the social-cognitive processes that, for most individuals, occur intuitively and without much conscious thought. So, for example, opportunities to discuss with the child specific instances of interpersonal behavior (conflict or positive interactions) and the step-by-step thought-feeling-behavior links may reveal for the parent a sense of the kind of impairments in processing social information that is now being suggested in clinical studies and experiences with children with RAD behavior.

As regards more formal interventions, it is notable that one study found sizable improvements in RAD and other problem behavior in a 7-year-old girl with suspected RAD [43]. What is noteworthy about this single case report is not the level of evidence it offers - which is minimal - but rather the notion that it may be helpful to consider a range of intervention options outside of those that have been offered so far. The point here is that it is far too easy to foreclose on any intervention model for RAD because so little is known about its treatment and the mechanisms of treatment.

Lastly, it is worth noting the important Bucharest Early Intervention Project [44]. Although it was not designed as an intervention for attachment disorder-related behavior (but rather for cognitive and social development more generally), the study used the RCT structure to examine the extent to which a home-rearing (i.e., foster) care setting was associated with positive cognitive gains over continued institutionalization. Children in the foster care setting exhibited significantly improved cognitive recovery than those children who stayed in an institutional setting; there was a further suggestion that the early removal may have been especially important. The application to attachment-related or other outcomes is unclear, but the study provides the rare example of an intervention to alter and test the impact of early rearing conditions on human development.

#### What is evidence-based?

Probably the most important lesson from the RAD debate and literature so far produced is the need for clinicians, trainees, and parents to have a clear sense of what constitutes evidence. Our current era of "evidence-based" practice places particular emphasis on evidence-base without defining what evidence is, or demonstrating that there are different levels of evidence. As regards the latter point, it is widely recognized that there are different levels of evidence, and that only higher level of evidence would garner clinical attention or imitation. So, for example, low levels of evidence, based on clinical reports provide very little basis for shaping clinical practice; the uncontrolled trial provides somewhat more, but is still well short of adequate. The gold standard RCT is probably the first point at which clinicians would take notice on a wide-scale, and the replicated RCT is probably the highest level of "actionable" evidence. To be sure, there are very few examples of RCTs that are replicated, particularly outside the group that initially formed the treatment. Fortunately, however, these practices are getting their due notice. How long it will be for other interventions to catch up to that level of evidence is unclear, but it is certainly a long-term goal. Until that time, there is a greater need for consumers of clinical science to attend to the basis for scientific claims of "evidence."

## Conclusion

Recent animal and human research findings show that there can be long-term effects of early adversity, such as caregiving deprivation. That does not mean that the human studies are yet mimicking what has been reported in the animal literature for many decades, however. The degree of individual differences shown consistently in human work has no obvious analogue in the animal work and remains perhaps the most impressive finding in human development. Moreover, with the noteworthy exception of the extreme case of institutional rearing, human studies have rarely been able to demonstrate that there is something particular to early attachment experiences that may have lasting effect. Progress in understanding genetics and neuroscience more broadly, along with progress in neuroscientific techniques, may yield insights that will benefit the study of attachment experiences and their role in mental health in young people and, more broadly, the interplay of biology and experience in human development. That progress will then, we hope, yield some directly applicable lessons concerning the role of timing in planning prevention and intervention strategies.

## Competing interests

The authors declare that they have no competing interests.
